# A Novel Cell Lysis Approach Reveals That Caspase-2 Rapidly Translocates from the Nucleus to the Cytoplasm in Response to Apoptotic Stimuli

**DOI:** 10.1371/journal.pone.0061085

**Published:** 2013-04-15

**Authors:** Alexander A. Tinnikov, Herbert H. Samuels

**Affiliations:** Department of Biochemistry and Molecular Pharmacology, New York University School of Medicine, New York, New York, United States of America; German Cancer Research Center, Germany

## Abstract

Unlike other caspases, caspase-2 appears to be a nuclear protein although immunocytochemical studies have suggested that it may also be localized to the cytosol and golgi. Where and how caspase-2 is activated in response to apoptotic signals is not clear. Earlier immunocytochemistry studies suggest that caspase-2 is activated in the nucleus and through cleavage of BID leads to increased mitochondrial permeability. More recent studies using bimolecular fluorescence complementation found that caspase-2 oligomerization that leads to activation only occurs in the cytoplasm. Thus, apoptotic signals may lead to activation of caspase-2 which may already reside in the cytoplasm or lead to release of nuclear caspase-2 to the extra-nuclear cytoplasmic compartment. It has not been possible to study release of nuclear caspase-2 to the cytoplasm by cell fractionation studies since cell lysis is known to release nuclear caspase-2 to the extra-nuclear fraction. This is similar to what is known about unliganded nuclear estrogen receptor-α (ERα ) when cells are disrupted. In this study we found that pre-treatment of cells with N-ethylmaleimide (NEM), which alkylates cysteine thiol groups in proteins, completely prevents redistribution of caspase-2 and ERα from the nucleus to the extra-nuclear fraction when cells are lysed. Using this approach we provide evidence that apoptotic signals rapidly leads to a shift of caspase-2 from the nucleus to the extra-nuclear fraction, which precedes the detection of apoptosis. These findings are consistent with a model where apoptotic signals lead to a rapid shift of caspase-2 from the nucleus to the cytoplasm where activation occurs.

## Introduction

Programmed cell death or apoptosis is a major pathway targeted in the treatment of various tumors by chemo- and radiation- therapies that is mediated by caspase activation. Mammalian cells express numerous caspases which have been categorized as “initiator” caspases (e.g. caspase-2, 8, 9, and 10), and “effector” caspases (e.g. caspase-3, 6, and 7) [1], [2], [3], [4]. The initiator caspases contain an extended N-terminal domain referred to as CARD (caspase-recruitment domain) or DED (death effector domain). CARD/DED acts to bring the initiator caspases in close proximity leading to a conformational change and oligomerization to form an activated caspase [3], [4]. This active initiator caspase then cleaves and activates effector caspases which leads to cleavage of a wide variety of protein components in the cell. The “initiator-effector” caspase cascade is best exemplified by the “extrinsic” pathway involving surface membrane death receptors (e.g. Fas, TNFα, and TRAIL) [4].

Apoptosis also occurs through an intrinsic pathway initiated by intracellular stress signals such as DNA damage and radiation [4]. The intrinsic pathway involves changes in permeability of the outer mitochondrial membrane (regulated by the Bcl-2 family of proteins) and the release of a number of mitochondrial proteins such as cytochrome c, AIF, Smac/DIABLO, and EndoG [4], [5], [6]. After release, AIF translocates to the nucleus and is thought to lead to cleavage of DNA into large fragments. The released cytochrome c complexes with the WD-40 repeats of Apaf-1 which leads to oligimerization of Apaf-1 which recruits procaspase-9 through its CARD domain [7]. This structure (apoptosome) activates procaspase-9, which then activates effector caspases such as caspase-3.

Although caspase-2 has been classified as an initiator caspase, until recently its role in apoptosis has been considered minor since caspase-2 knockout mice exhibit only minor phenotypic changes [8]. In addition, unlike caspase-8 or -9, activated caspase-2 does not appear to cleave known effector caspases leading to a proteolytic cascade [9], [10], [11]. Although caspase-2 had long been thought to act downstream of mitochondria, recent studies indicate an important role for caspase-2 in stress-induced mitochondrial permeability. Caspase-2-mediated changes in mitochondrial permeability can occur without activation of caspase-9, although these changes in mitochondrial permeability lead to the release of cytochrome c, which activates caspase-9 which then activates caspase-3 [4], [10], [12], [13], [14].

Several years ago we identified a nuclear hormone receptor co-activator which we refer to as Nuclear Receptor Interacting Factor 3 (NRIF3). Expression of NRIF3 specifically and rapidly (within 5 h) leads to caspase-2-dependent apoptosis in a wide variety of breast cancer cell lines (Estrogen Receptor positive or negative) but not other cell types [15], [16], [17]. Evidence that this is mediated by caspase-2 comes from the finding that stable knockdown of caspase-2 by RNAi abrogates the ability of NRIF3 to induce apoptosis of breast cancer cells [15]. This effect of NRIF3 on mediating apoptosis of breast cancer cells is independent of its role as a nuclear receptor co-activator. NRIF3 mediates apoptosis by binding to a transcriptional repressor which we identified (DIF-1) [17] and reverses the ability of DIF-1 to repress the pro-apoptotoc gene, FASTKD2 (FAST kinase domains 2) [18]. Although FASTKD2 is only derepressed by NRIF3 in breast cancer cell lines, transiently expressed FASTKD2 leads to caspase-2 dependent apoptosis in other cell types (e.g. HeLa cells) [18].

Unlike other caspases, caspase-2 appears to localize to the cell nucleus [14], [19], [20]. However a number of reports indicate localization to the cytosol, golgi or mitochondria [9], [19], [21], [22], [23], [24]. These different findings on the sub-cellular localization of endogenous caspase-2 may be dependent on the cell type and/or the specificity of the antibodies used for immumocytochemistry. Evidence that caspase-2 is a nuclear protein comes from immunofluorescent studies of endogenous caspase-2 or imaging of GFP-caspase-2 chimeras expressed in cells [14], [19], [20]. Analysis of GFP-fused to the N-terminus (GFP-caspase-2) or C-terminus of caspase-2 (caspase-2-GFP) supported the notion that nuclear caspase-2 can signal to the mitochondria *via* cleavage of BID to tBID which then leads to changes in mitochondrial membrane permeability and release of cytochrome c [14]. In contrast, a more recent study using “bimolecular fluorescent complementation” to identify caspase-2 oligomerization which leads to activation suggested that this process takes place in the cytoplasm and not the nucleus [25], [26]. This raises the possibility that a pro-apoptotic signal leads to nuclear caspase-2 release into the cytoplasm where activation occurs.

Although studies on the cell distribution of caspase-2 confirmed nuclear localization of endogenous caspase-2 or GFP-caspase-2 [14], [20], cytoplasmic caspase-2 or GFP-caspase-2 are identified after subcellular fractionation [14]. Thus, cell lysis studies may not accurately reflect the cell distribution of caspase-2 since redistribution of nuclear caspase-2 may occur upon cell lysis. An example of such artifactual redistribution upon cell lysis was previously documented for the human estrogen receptor- (ERα ) [27]. Although numerous cell fractionation studies over many years indicated that in the absence of estrogen agonists ERα localizes to the extra-nuclear fraction, subsequent immunocytochemistry studies indicated that unliganded ERα was a nuclear protein [27].

As described in this study, in the course of examining the role of caspase-2 in the apoptotic response mediated by FASTKD2 in HeLa cells, we found that caspase-2 is rapidly released from the nucleus to the extra-nuclear fraction upon cell lysis. Exportin 1 (CRM1), an evolutionarily conserved receptor for the nuclear export signal of proteins, has been shown to be the cellular target of leptomycin B, a nuclear export inhibitor [28]. In yeast, leptomycin B was shown to act by modifying cysteine 529 in CRM1 (Cys-529) which acts to inhibit its role in nuclear export of proteins [28]. N-ethylmaleimide (NEM), an alkylating agent that is rapidly taken up by cells [29] and modifies cysteine thiol groups in proteins, also blocked nuclear export in yeast, presumably by modifying Cys-529 [28].

In this regard, we examined the cell distribution of caspase-2 after adding NEM to cells prior to cell lysis. Pre-incubation of cells with NEM completely blocks redistribution of nuclear caspase-2 to the extra-nuclear fraction upon cell fractionation. Surprisingly, pre-incubation of cells with high concentrations of leptomycin B was without effect. In similar studies, NEM was found to block ERα redistribution from the nucleus to the extra-nuclear fraction upon cell lysis. Although the precise mechanism by which NEM prevents redistribution of caspase-2 or ERα has not been defined, NEM serves as a useful tool to study the cell distribution and possible activation of caspase-2 using cell fractionation under physiologic conditions and during caspase-2-dependent apoptosis.

## Materials and Methods

### Plasmids

Vectors expressing GFP-Caspase-2 wild-type and the inactive GFP-caspase-2 mutant (Cys303Ser change in the catalytic site) were obtained from Toshiyuki Miyashita, Department of Genetics, National Children’s Medical Research Center, Tokyo, Japan [20]. Both are human caspase-2 clones. A pCDNA vector expressing FLAG-tagged human ERα was obtained from Michael Garabedian, NYU Medical Center.

### Cell Culture, DNA Transfection, and Cell Fractionation

HeLa cells were routinely maintained in DMEM containing 10% bovine calf serum supplemented with glutamine and antibiotics. Cells were seeded in 24, 12 or 6-well plates at least 24 h prior to transfection. The cells at 60–80% confluency were transfected with indicated plasmid(s) using Lipofectamine 2000 (Invitrogen). For studies with GFP-caspase-2 chimeras or FLAG-ERα, cells were harvested 15–20 h after transfection.

Cells were treated with the indicated concentrations of NEM or iodoacetic acid (IAA) in serum free DMEM for the times indicated. Untreated cells served as a control. The cells were then washed with ice-cold isotonic saline and lysed in ice-cold lysis buffer (50 mM Tris-HCl (pH 7.4), 150 mM KCl, 0.5% Triton X-100 with Roche mini-complete protease inhibitor) by repeated vortexing at maximal setting and keeping the cells on ice for 10 min. Lysing cells with Triton X-100 at isotonic salt concentrations has been shown to be a rapid and effective way to isolate purified nuclei [30]. The lysates were centrifuged for 10 min at 5,000 g, the extra-nuclear faction was transferred to a new tube and the nuclear pellets were washed with lysis buffer. The nuclear pellets were boiled in 1x SDS loading buffer, vortexed and centrifuged at 5,000 g for 5 min and the supernatant representing nuclear extract was collected. NEM-treated cells do not adhere to the culture flasks as efficiently as untreated cells and, as a result, we lose some of the NEM-treated cells during the isotonic saline washing procedure. Protein was determined in the derived extra-nuclear fractions by the Bradford Procedure (Pierce) to correct for differences in cell material isolated between the control and NEM-treated cells. These differences were used to correct for the amount of nuclear extract analyzed. In studies comparing the level of caspase-2 in the extra-nuclear (lysate) and nuclear fractions, identical cell equivalents of material were analyzed by Western blotting. For example, if 30% of the total extra-nuclear fraction was used, this would be compared with 30% of the total nuclear extract. Similar studies were also carried out in cells pre-incubated with 200 uM zVDVAD-fmk for 15 h (caspase-2) or 100 nM leptomycin B for 2 h (caspase-2 and FLAG-ERα ). In the figures in this paper the term “Lysate” refers to the extra-nuclear fraction isolated with Triton X-100.

**Figure 1:**
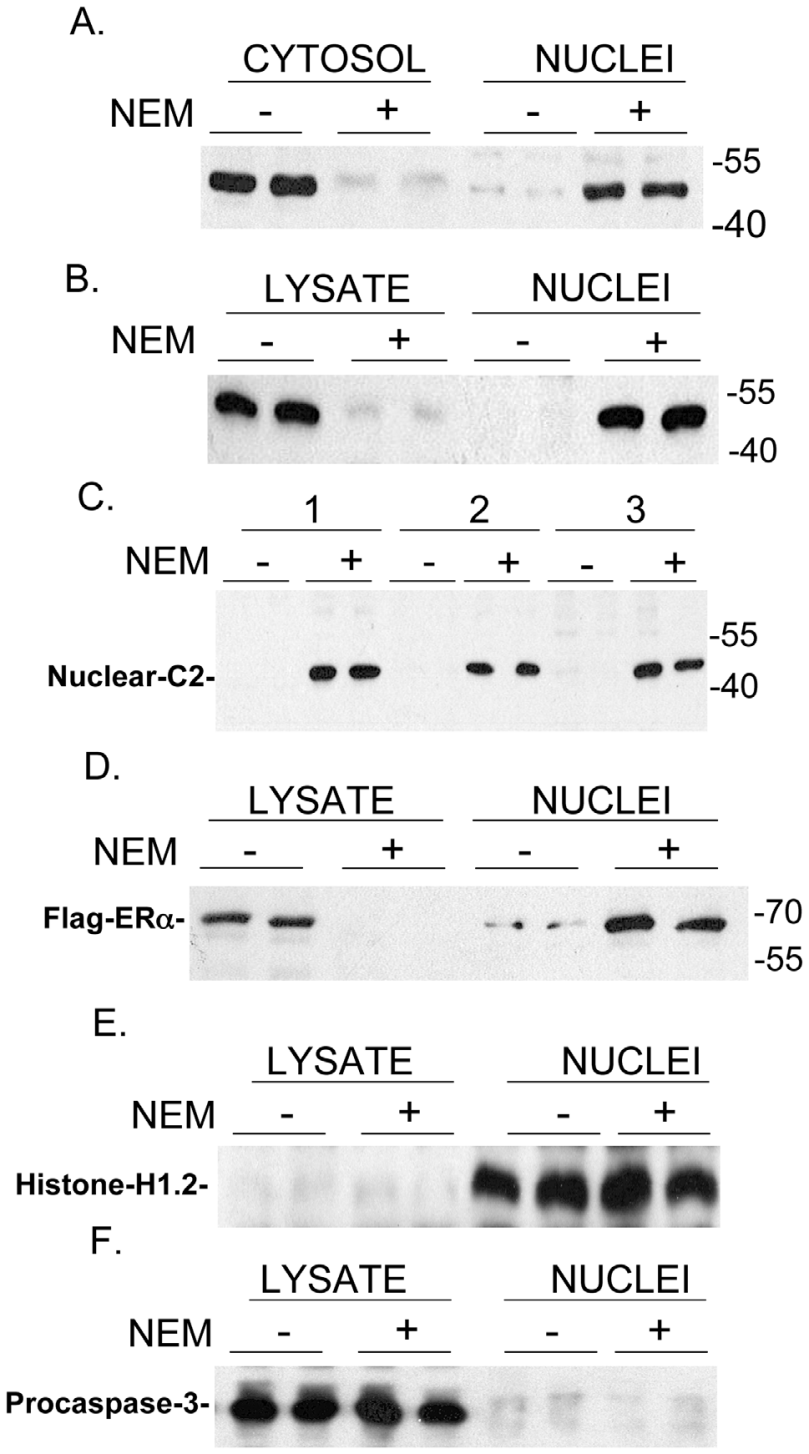
Effect of N-Ethylmaleimide (NEM) on the distribution of endogenous caspase-2 (C2) (Panels A–C) and FLAG-ERα (Panel D) between nuclear and extra-nuclear (cytosol or lysate) cell fractions. (A) Caspase-2 in cytosol and nuclei of cells without (−) or with (+) NEM (20 mM) pre-treatment for 10 min prior to cell fractionation using hypotonic buffer without detergent. (B) Caspase-2 in the lysate and nuclei of cells without (−) or with (+) NEM (7.5 mM) pre-treatment for 10 min prior to cell lysis with buffer containing 0.5% Triton X-100. (C) Nuclear Caspase-2 in cells without (−) or with (+) NEM (7.5 mM) pre-treatment for 10 min prior to lysis using the following conditions: 1) after lysis in cell culture plates (lysis buffer added directly to the plate wells), 2) cells in suspension collected in an Eppendorf tube were instantly frozen in dry ice/ethanol, transferred to ice bath and ice-cold lysis buffer with Triton X-100 was immediately added to the frozen pellets, 3) cells in suspension collected in an Eppendorf tube at room temperature and room temperature lysis buffer with 0.5% Triton X-100 was added to the pellets. (D). FLAG-tagged ERα in the lysate and nuclei of cells without (−) and with (+) NEM (20 mM) pre-treatment for 10 min prior to cell lysis with buffer containing 0.5% Triton X-100. The numbers on the right of the panels reflect the gel migration of the 40 kDa, 55 kDa, and 70 kDa protein markers. To ensure that our cell lysis procedure actually reflects nuclear and extra-nuclear (LYSATE) fractions, Western blotting studies examined for Histone H1.2 as a nuclear marker (E) and Procaspase-3 as an extra-nuclear marker (F) [31]. Cell lysis was carried out using the conditions given in (B) above.

The purity of the nuclear and extra-nuclear fractions was assessed by Western blotting for a nuclear marker (Histone H1.2) and an extra-nuclear marker (Procaspase-3). No H1.2 was found in the extra-nuclear fraction (Abcam antibody ab17677**)** and no Procaspase-3 (Santa Cruz sc7148) was found in the nuclear fraction (see Figure 1 panels E and F). Although, caspase-3-p12 and caspase-3-p17 are found in the nucleus, Procaspase-3 is only found in the extra-nuclear fraction in normal cells and in cells undergoing apoptosis [31].

For studies on the accumulation of caspase-2 in the extra-nuclear fraction after treating cells with H_2_O_2_ or etoposide (in serum free DMEM), the cells were incubated for the indicated times with H_2_O_2_ or etoposide concentrations known to induce apoptosis [15]. After H_2_O_2_ or etoposide incubation the medium was replaced with fresh serum free medium containing 20 mM NEM for 10 min before cell lysis.

For studies with FLAG-tagged ERα, prior to and after transfection, cells were incubated with DMEM containing 10% (v/v) AG1x8 resin/charcoal treated bovine calf serum as previously described [32]. This treatment efficiently extracts phenols and aromatic compounds from serum and removes over 95% of estrogen and other estrogen receptor agonists from the serum, thus allowing for an assessment of the cell distribution of ligand-unbound FLAG-tagged ERα.

In one experiment, a detergent-free cell fractionation method was used. After washing with isotonic saline, cells were collected with ice cold Cell Extraction Buffer (20 mM Tris, 10 mm KCl, 1.5 mM MgCl, 1 mM EDTA, 1 mM EGTA) and kept on ice and allowed to swell for 15 min. Cells were vortexed at maximal setting and then frozen in dry ice/ethanol and after thawing syringed 10 times with 1 ml tuberculin syringe with a 27 gauge needle. The homogenate was centrifuged at 5,000 g to obtain cytosol (supernatant) and the nuclear pellet. The nuclear pellet was washed twice with Cell Extraction Buffer and the nuclear proteins were extracted by boiling in 1x loading buffer as described above.

After SDS-PAGE fractionation, proteins were transferred to a nitrocellulose membrane (NitroBind, GE Water and Process Technologies), blocked with BSA and probed with caspase-2 rat monoclonal antibody (11B4) (Alexis-Enzo Life Sciences) or with FLAG antibody (M2) (Sigma-Aldrich) to detect FLAG-ERα. Histone H1.2 was detected with antibody from Abcam (ab17677) and Procaspase-3 detected with antibody from Santa Cruz (sc7148).

## Results

### Effect of NEM and Iodoacetic Acid on Cell Distribution of Caspase-2 Upon Cell Lysis

**Figure 1** indicates that pre-incubation of cells with NEM (7.5 mM or greater) for 10 min prior to cell fractionation blocks redistribution of caspase-2 from the nucleus to the extra-nuclear fraction (designated as cytosol or lysate). In **Figure 1A** cells were disrupted in hypotonic buffer without Triton X-100 while in **Figure 1B** cells were lysed in the presence of 0.5% Triton X-100. Both procedures gave similar results. It should be noted that caspase-2 in these and other studies in this paper reflect the size of Procaspase-2 (∼50 kDa) and we detected no evidence for caspase-2 cleavage in these studies. In **Figure 1C** cells were pre-incubated with or without NEM for 10 min and were then lysed under three different conditions. Condition 1 reflects cells collected with lysis buffer as in **Figure 1B**. In Condition 2 cells were released from the cell culture plates with buffer containing EDTA and were then flash frozen in dry ice/ethanol to attempt to “trap” caspase-2 and then lysed. In Condition 3 the suspended cells were lysed at 25°C. Each lysis condition gave identical results; pre-incubation with NEM completely prevented loss of nuclear caspase-2. Importantly, these findings reflect an effect of pre-incubation of cells with NEM since addition of NEM to the lysis buffer used for cell fractionation showed only extra-nuclear caspase-2 (not shown).

ERα is known to localize to the cell nucleus in the absence of agonist as assessed by immunocytochemistry but is found predominantly in the cytosol when cells are lysed [27]. This altered cell distribution of ERα upon cell lysis has been shown to be an artifact of cell lysis [27]. Thus, we examined the effect of NEM on the cell distribution of ERα (**Figure 1D**). HeLa cells were transfected with a vector expressing FLAG-ERα. Fifteen h later the cells were incubated with or without 20 mM NEM for 10 min. Cells were lysed and the cell distribution of FLAG-ERα determined by Western blotting with FLAG-M2 antibody. NEM pre-incubation resulted in the same distribution of FLAG-ERα as endogenous caspase-2 supporting the notion that our NEM results with caspase-2 reflect its cell distribution prior to cell lysis. We also reproduced the effect of NEM on the distribution of caspase-2 and FLAG-ERα in studies using T-47D breast cancer cells (not shown). To ensure that our cell lysis procedure actually reflects nuclear and extra-nuclear (Lysate) fractions, Western blotting studies examined for Histone H1.2 as a nuclear marker (**Figure 1E**) and Procaspase-3 (∼35 kDa) as an extra-nuclear marker (**Figure 1F**) [31]. Procaspase-3, unlike its cleavage products (p12 ad p17), is exclusively found in the extra-nuclear fraction [31].

**Figure 2:**
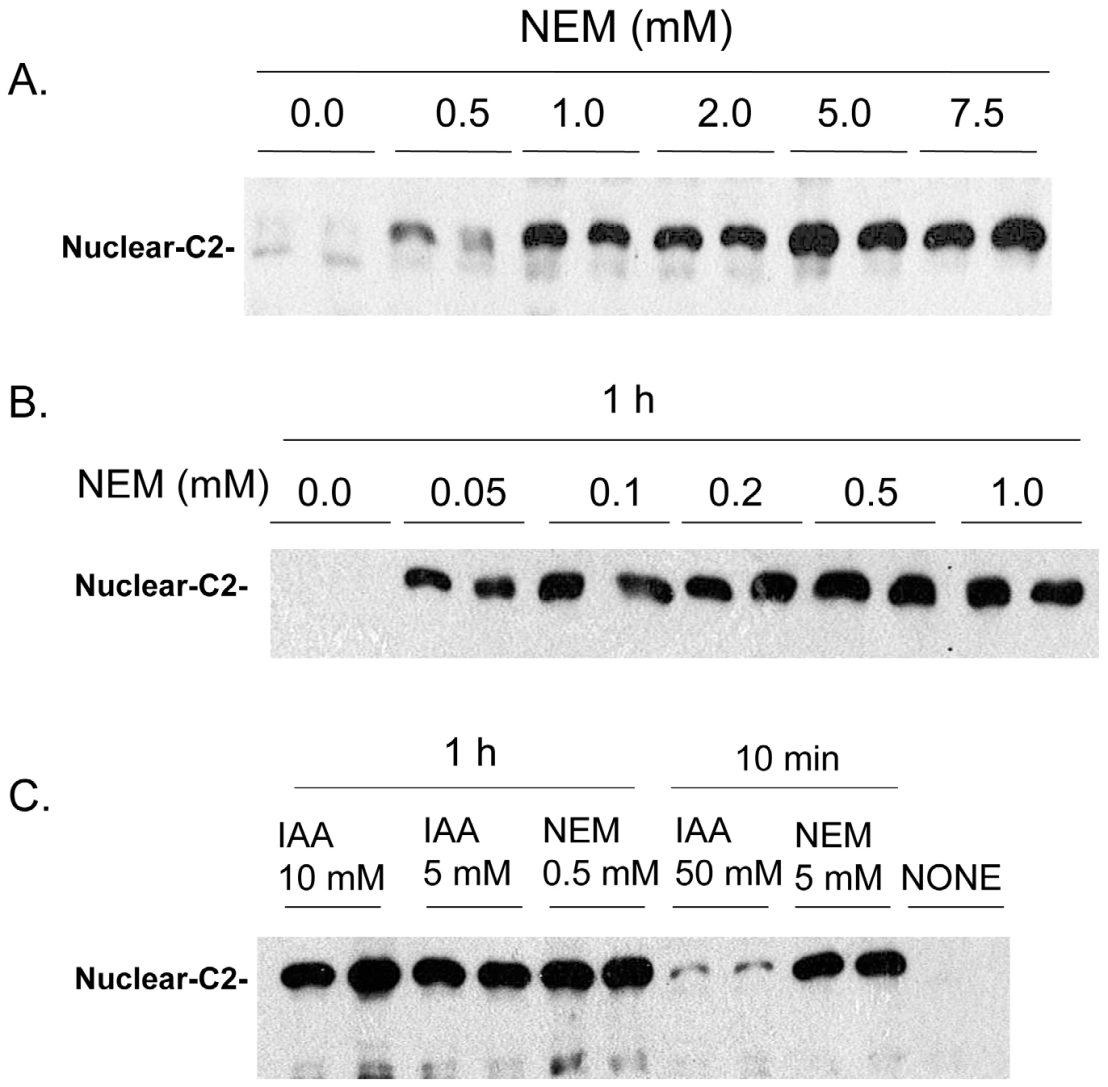
Effect of NEM and IAA concentrations on endogenous nuclear caspase-2 levels. (A) Cells were pretreated with the indicated concentrations of NEM for 10 min before lysis. (B) Effect of NEM concentrations on nuclear caspase-2 levels. Cells were pre-treated with NEM as indicated for 1 h before lysis. (C) Effect of 10 min or 1 h pre-treatment with the indicated concentrations of Iodoacetic Acid (IAA) or NEM on caspase-2 levels in the nuclear fraction.

**Figure 3:**
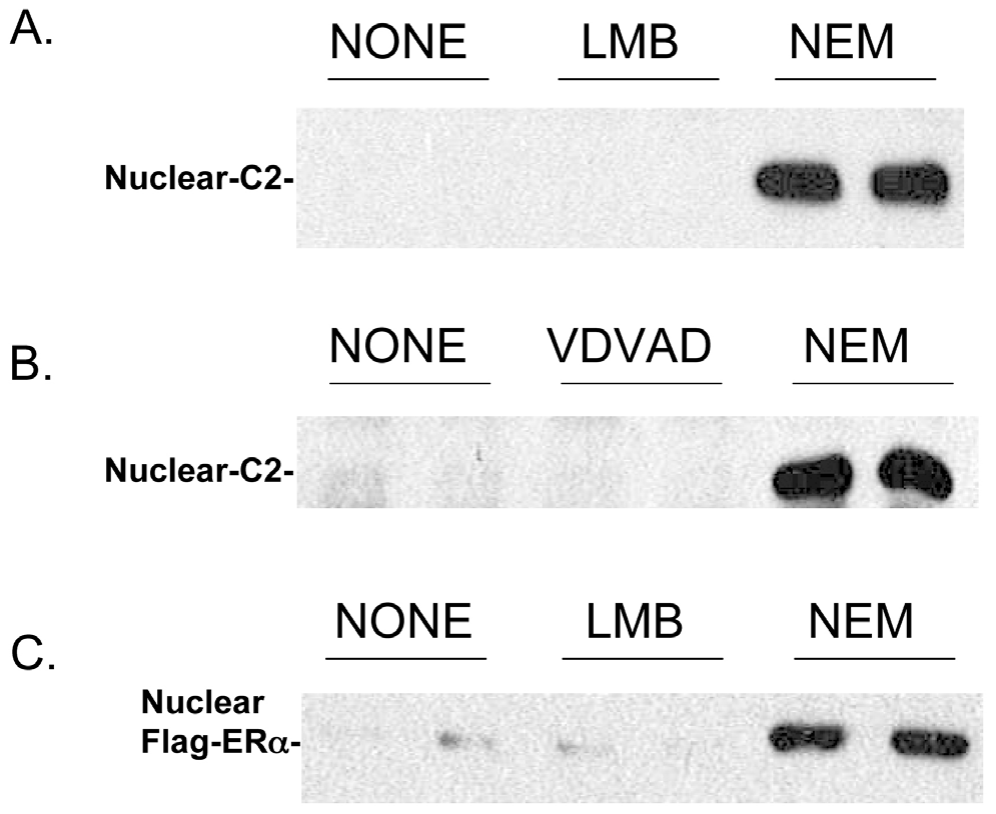
Effect of leptomycin B on nuclear caspase-2 and FLAG-ERα levels. (A) Cells were incubated with NEM (20 mM) for 10 min or with high levels of leptomycin B (100 nM) (LMB) for 2 h. Parallel-untreated cells (NONE) served as a control. The cells were then lysed and the level of endogenous nuclear caspase-2 determined by Western blotting. (B) Effect of zVDVAD-fmk on nuclear caspase-2 levels. Cells were incubated with a high level of zVDVAD-fmk (200 uM) for 15 h while parallel cells incubated with zVDVAD-fmk also received 20 mM NEM for 10 min prior to harvesting. Parallel-untreated cells (NONE) served as a control. The level of caspase-2 in the nuclear fraction was determined by Western blotting. (C) Similar to the study described in (A) except that the effect of leptomycin B and NEM was examined on nuclear FLAG-ERα levels 15 h after FLAG-ERα expression.

**Figure 2** Illustrates the effect of NEM concentration and cell incubation times on the level of nuclear caspase-2 after cell lysis. **Figure 2A** illustrates the effect of a 10 min pre-incubation of cells with the indicated NEM concentrations on the level of nuclear caspase-2 after cell lysis. Maximal levels of nuclear caspase-2 are found with 5 mM NEM or greater. Lower concentrations of NEM are similarly effective if the cells are pre-incubated for 1 h with a maximal effect seen at 0.5 mM (**Figure 2B**). We presume that NEM acts by modifying one or more essential cysteines in proteins that somehow influence caspase-2 nuclear retention upon cell lysis. To further support that possibility we compared nuclear caspase-2 levels in cells pre-incubated with NEM or iodoacetic acid (IAA), which also covalently modifies thiol groups found in cysteines (**Figure 2C**). Cells pre-incubated for 1 h with 0.5 mM NEM or 5 mM IAA resulted in similar nuclear caspase-2 levels after cell lysis. However, with a 10 min pre-incubation, 5 mM NEM was more effective than 50 mM IAA suggesting that the difference in efficacy of NEM and IAA relates to the kinetics of cell uptake. We also explored the effect of pre-incubation of 100 nM leptomycin B (LMB) for 2 h on the cell distribution of endogenous caspase-2 (**Figure 3A**) as well as expressed FLAG-ERα (**Figure 3C**) after cell lysis and found no effect suggesting that NEM and IAA act independent of modification of Exportin 1. In addition, pre-incubation with high concentrations of zVDVAD-fmk (200 uM for 15 h), which inactivates activated caspase-2, was without effect on the cell distribution of caspase-2 (**Figure 3B**).

### Comparison of the Cell Distribution of Wild-type and a Caspase-2 Mutant with Modification of an Essential Cysteine in the Catalytic Site after Cell Lysis

**Figure 4:**
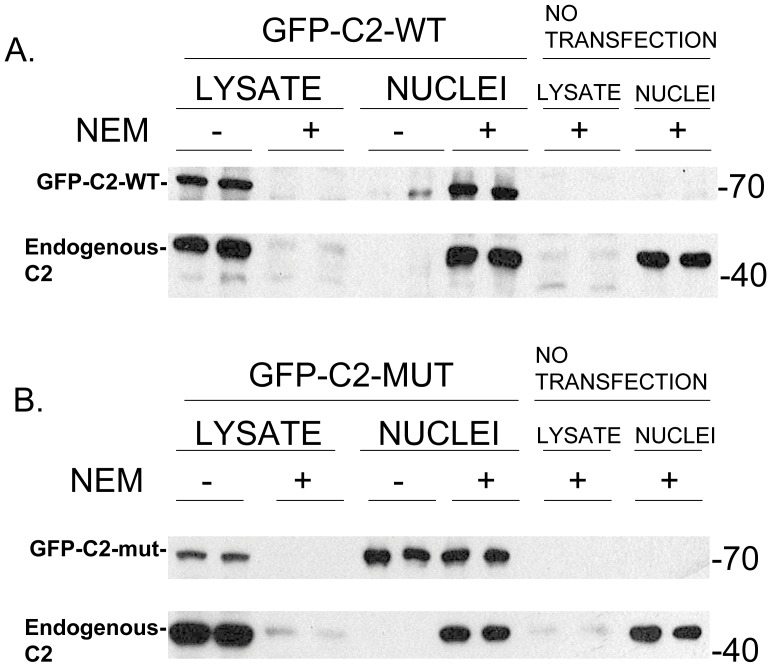
Effect of NEM on the cell distribution of wild-type and mutant caspase- 2. Shown is the distribution of wild-type GFP-Caspase-2 (GFP-C2-WT) (A), and mutant GFP-caspase-2 (GFP-C2-MUT) (B) between nuclear and extra-nuclear fractions of cells without (−) or with (+) NEM (7.5 mM) pre-treatment for 10 min prior to cell lysis. Also shown in both panels is the distribution of endogenously expressed caspase-2 in response to NEM pre-treatment in the same cells. The numbers on the right of the panels reflect the gel migration of the 40 kDa and 70 kDa protein markers.

Since human caspase-2 contains an essential cysteine in its catalytic site (Cys303) we explored the possibility that modification of this cysteine by NEM might somehow explain, in part, the change in cell distribution upon cell lysis. HeLa cells were transfected with vectors expressing GFP-caspase-2 wild-type (GFP-C2-WT) or GFP-caspase-2 with a modified cysteine (Cys303Ser) [20] in the active site (GFP-C2-mut). The amount of vector used in the transfections were chosen to express equal or lower amounts of GFP-caspase-2 than endogenous caspase-2. **Figure 4A** illustrates the effect of NEM pre-incubation on the cell distribution of GFP-caspase-2 wild-type and endogenous caspase-2. The effect of NEM was identical for both GFP-caspase-2 wild-type and endogenous caspase-2; NEM pre-incubation prevented leakage into the extra-nuclear fraction after cell lysis. In contrast, the cell distribution of the GFP-caspase-2 mutant differs from that of endogenous caspase-2 (**Figure 4B**). Without NEM pre-treatment, most of the GFP-caspase-2 mutant remains in the nucleus after cell lysis with very little effect of NEM on its level in the nuclear fraction. However, a small amount of the GFP-caspase-2 mutant is found in the extra-nuclear lysate fraction and this is prevented with pre-treatment of the cells with NEM. In contrast, nuclear localization of endogenous caspase-2 in the same cells after cell lysis is completely dependent on NEM pre-incubation. We have consistently found these results when comparing wild-type and the mutant caspase-2 suggesting that NEM acts, in part, to prevent redistribution of caspase-2 by modifying an essential cysteine in the caspase-2 catalytic site.

### Caspase-2 Rapidly Accumulates in the Extra-nuclear Fraction in Response to Apoptotic Stimuli

**Figure 5:**
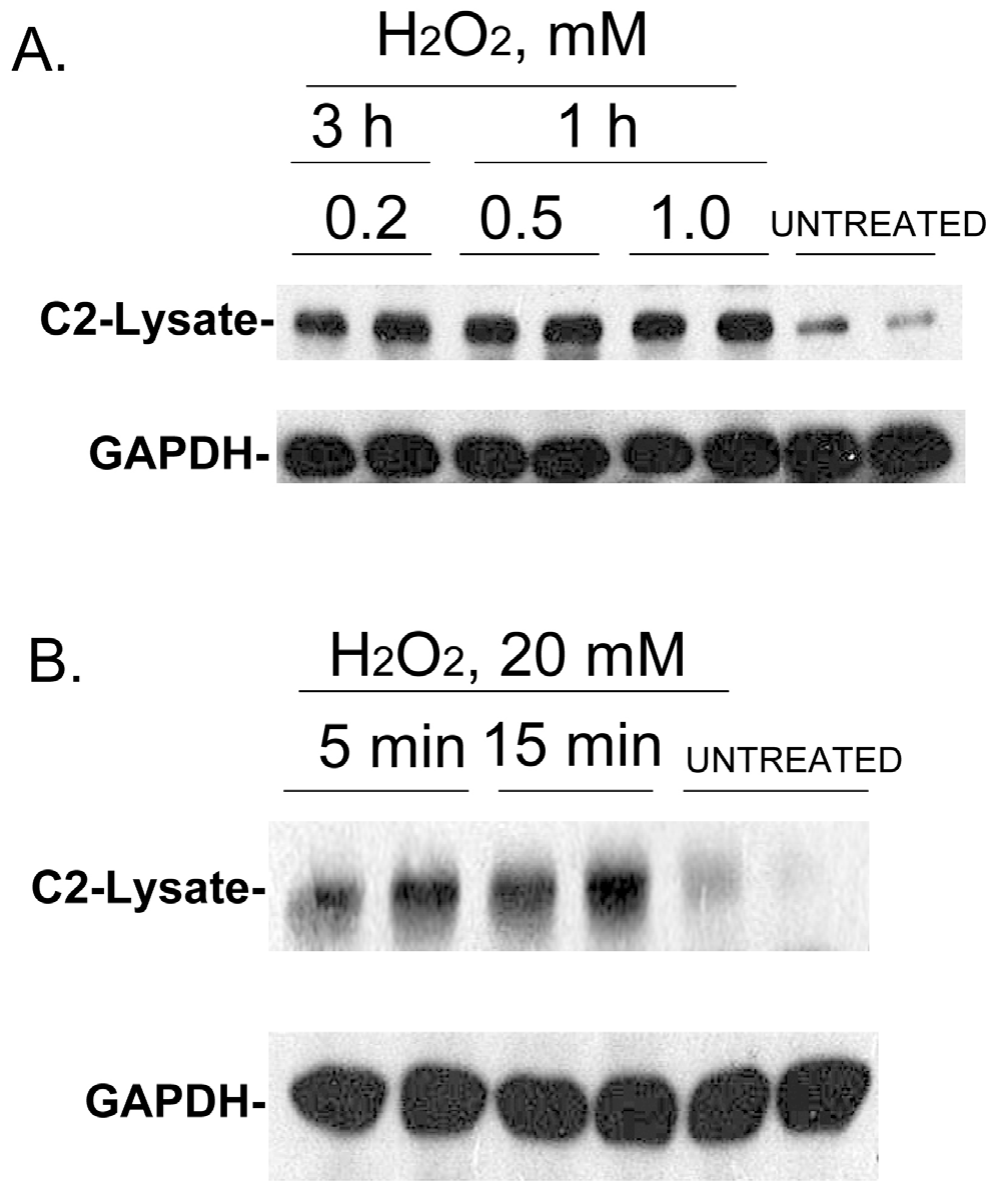
H_2_O_2_ incubation leads to rapid accumulation of caspase-2 in the extra-nuclear fraction of lysed cells. (A) Caspase-2 in the extra-nuclear fraction (designated as lysate) after treating cells for 1 and 3 h with concentrations of H_2_O_2_ known to induce apoptosis. (B) Caspase-2 levels in the extra-nuclear fraction after treatment with 20 mM H_2_O_2_ for 5 or 15 min. After H_2_O_2_ incubation the medium was replaced with serum free medium containing 20 mM NEM for 10 min before lysis.

It has been suggested that caspase-2 mediates changes in mitochondrial permeability from its location in the cell nucleus through cleavage of BID to tBID [14]. However, a recent study using bimolecular fluorescence complementation using “split” fragments of the “Venus” version of Yellow Fluorescent Protein supports the notion that caspase-2 activation occurs exclusively in the cytoplasm [25], [26]. Using NEM to “trap” caspase-2 in the nuclear or extra-nuclear fraction during cell lysis in cells incubated with concentrations of hydrogen peroxide (H_2_O_2_) that leads to apoptosis [15] supports this proposal. HeLa cells were treated with H_2_O_2_ at the concentrations and times indicated in **Figure 5** and the cells were pre-treated with 20 mM NEM for 10 min before lysis. Shown in **Figure 5A and B** is accumulation of full-length endogenous caspase-2 (∼50 kDa) in the extra-nuclear fraction in response to H_2_O_2_ incubation. Accumulation of caspase-2 in the extra-nuclear fraction is rapid and is detected within 1 h with 0.2–1 mM H_2_O_2_ (**Figure 5A**) and as early as 5 min with 20 mM H_2_O_2_ (**Figure 5B**).

**Figure 6:**
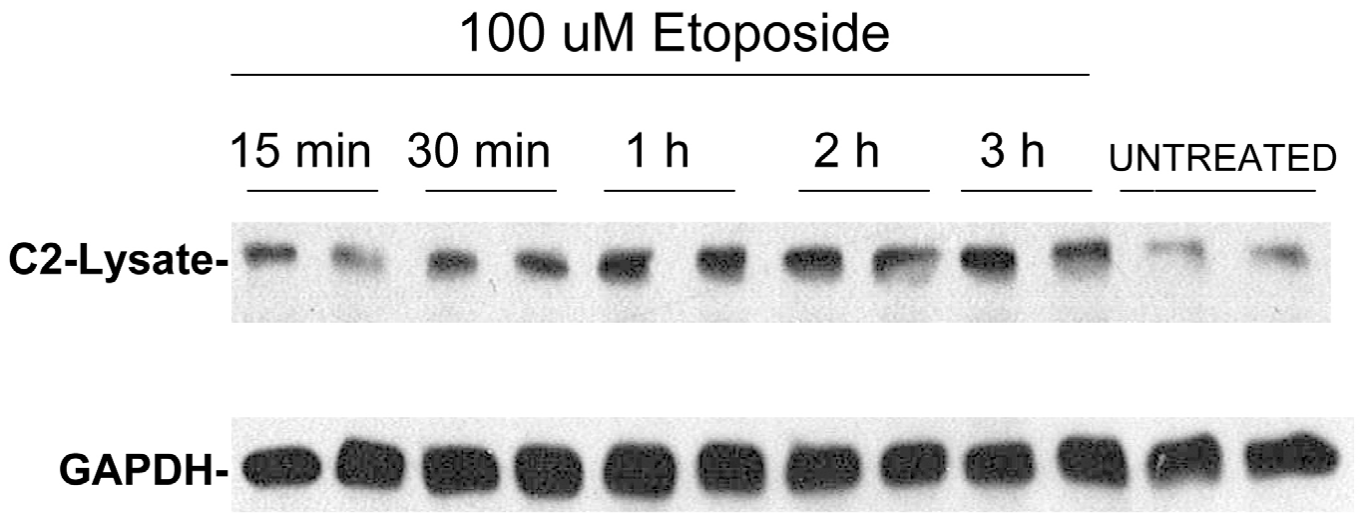
Etoposide incubation leads to rapid accumulation of caspase-2 in the extra-nuclear fraction of lysed cells. The results show the accumulation of caspase-2 in the extra-nuclear fraction (designated as lysate) after treating cells for 15 min to 3 h with 100 uM etoposide. After etoposide incubation the medium was replaced with serum free medium containing 20 mM NEM for 10 min before lysis.

Since H_2_O_2_ leads to apoptosis through oxidative stress we sought to determine if other apoptotic stimuli lead to the rapid accumulation of extra-nuclear caspase-2. Etoposide initiates DNA damage mediated apoptosis through mechanisms which include activation of caspase-2. **Figure 6** illustrates a study which examined the accumulation of extra-nuclear caspase-2 in response to the indicated concentrations and incubation times of etoposide followed by pre-treatment with 20 mM NEM for 10 min before cell lysis. Like H_2_O_2_ incubation, exposure of HeLa cells to etoposide leads to the rapid accumulation of full-length extra-nuclear caspase-2 and is detected within 30 min after incubation with 100 uM etoposide. Interestingly, this accumulation of caspase-2 in the extra-nuclear fraction occurs hours before the detection of apoptosis by TUNEL or other assays [15], [33], [34], [35].

In **Figures 5** **and** **6** the caspase-2 released in the extra-nuclear fraction in response to apoptotic stimuli represents only about 5% of total cell caspase-2. This is based on comparing the Western blotting exposure times to achieve caspase-2 bands of similar intensity using the same proportions of nuclear and extra-nuclear fractions. Although we attempted to verify the increase in extra-nuclear caspase-2 in response to apoptotic stimuli using immunofluorescence, we were not able to confirm this. However, this would likely be difficult to assess because only about 5% of caspase-2 accumulates in the extra-nuclear fraction and the released caspase-2 would be diluted in the extra- nuclear compartment.

## Discussion

Caspase-2 is the only caspase known to be localized to the nucleus and contains a well-characterized nuclear localization signal (NLS) [14], [36]. Although GFP-caspase-2 chimeras appear to only localize to the nucleus (both wild-type and a catalytic site mutant) [20], immumocytochemistry studies suggest that caspase-2 may also localize to the cytosol and the golgi [22], [23], [24], [37]. Whether these differences reflect the *in situ* distribution of caspase-2 or represent differences due to off-target specificity of the antibodies used in these studies has not been rigorously determined. Using immunofluorescence, Paroni et al. [14] reported that endogenous caspase-2 was exclusively a nuclear protein as was caspase-2 expressed with GFP fused to its C-terminus. Surprisingly, in parallel cell fractionation studies a substantial amount of caspase-2 or caspase-2-GFP was found in the extra-nuclear fraction which led the authors to conclude that caspase-2 dissociates from the nucleus during cell fractionation [14]. Mutation of the caspase-2 NLS resulted in a predominant cytoplasmic localization of the enzyme which leads to apoptosis indicating that caspase-2 need not be nuclear localized to generate an apoptotic response [14]. Bouchier-Hays et al. [25], [26] recently used bimolecular fluorescence complementation to study caspase-2 oligomerization (the initial step in activation) in real time and found that, in response to a number of apoptotic stimuli, oligomerization was detected in the cytoplasm rather than the nucleus [26]. Caspase-2 activation in the cytoplasm may reflect cytoplasmic localized caspase-2 or through a signaling mechanism where caspase-2 is released from the nucleus to the cytoplasm where the caspase is activated.

In studying caspase-2 localization by cell fractionation in HeLa cells (as well as T-47D cells) we found that upon cell lysis, with or without Triton X-100, all of the caspase-2 was found in the extra-nuclear fraction. We found that pre-incubation of cells for just 10 min with NEM (**Figure 1**) completely prevents redistribution of nuclear localized caspase-2 to the extra-nuclear fraction. These findings reflect an effect of pre-incubation of cells with NEM since addition of NEM to the lysis buffer used for cell fractionation showed only extra-nuclear caspase-2. Similar results were found for ERα which is known to artifactually transfer from the nucleus to the cytoplasm in the absence of ligand when cells are lysed [27]. We considered that NEM might act by alkylating an essential cysteine in Exportin 1 involved in nuclear export of proteins. However, pre-incubation of cells with high concentrations of the Exportin 1 inhibitor leptomycin B did not prevent the redistribution of nuclear caspase-2 or FLAG-ERα to the extra-nuclear fraction (**Figure 3**). Previous Immunofluorescence studies found that incubation of MCF-7 cells with leptomycin B resulted in an increase in the steady-state levels of nuclear ERα [38]. The finding that leptomycin B pre-incubation does not lead to nuclear retention of FLAG-ERα (**Figure 3**), further supports the notion that, as with caspase-2, Exportin 1 does not play a role in the nuclear to cytoplasmic redistribution of these proteins upon cell lysis.

Although we don’t know how NEM acts to retain caspase-2 in the nucleus when cells are lysed, we assume NEM modifies one or more cysteines in caspase-2 or in factors involved in the nuclear pore machinery in some way allowing for nuclear retention. Consistent with this notion is that similar results are found using IAA which also irreversibly modifies cysteines in proteins. This effect of NEM was found for endogenously expressed caspase-2 as well as transiently expressed wild-type GFP-caspase-2. Intriguingly, we have consistently found that GFP-caspase-2 with a mutation in an essential cysteine (Cys303Ser) in the catalytic site remains predominantly in the nucleus when cells are lysed suggesting that NEM acts, in part, by modifying that cysteine. This raises the possibility that somehow cell fractionation leads to activation of caspase-2, which then distributes from the nucleus to the extra-nuclear fraction. This does not seem likely, however, since cells pre-incubated with very high concentrations of zVDVAD-fmk (200 uM), which is known to irreversibly inactivate the catalytic site of active caspase-2, does not lead to retention of nuclear caspase-2 when the cells are lysed (**Figure 3**).

After microinjection of GFP-caspase-2 into cells, immunocytochemistry identified that nuclear caspase-2 eventually localizes to the cytoplasm [14]. This was considered to reflect a late apoptotic response that occurs after caspase-2 activation in the nucleus [14]. However, studies using bimolecular fluorescence complementation suggest that in response to apoptotic stimuli caspase-2 oligomerizes and is activated in the cytoplasm rather than the nucleus [26]. We used our findings with NEM to examine whether nuclear caspase-2 is released into the extra-nuclear fraction upon apoptotic stimuli. **Figure 5** illustrates such a study with H_2_O_2_ which is known to lead to apoptosis in HeLa cells [15]. H_2_O_2_ incubation rapidly (within 5 to 15 min) leads to the extra-nuclear accumulation of full-length caspase-2 which occurs prior to the time that apoptosis occurs as previously determined by TUNEL assay [15]. **Figure 6** shows similar findings for etoposide-mediated apoptosis in response to DNA damage. Caspase-2 rapidly accumulates in the extra-nuclear fraction and is maximal within 30 min to 1 h of exposure to 100 uM etoposide.

Recent studies have indicated that in addition to acting as an initiator caspase, caspase-2 plays an important role in cell-cycle checkpoint regulation and tumor suppression [10], [39] as well as DNA-damage induced expression of p21 possibly through enhanced translation of the protein [40]. Interestingly, the tumor suppression effect of caspase-2 in the mouse requires an active catalytic site [41]. In addition, a recent study using mouse embryo fibroblasts (MEFs) from caspase-2 knockout mice found that MEFs lacking caspase-2 display an increase in DNA damage, aneuploidy and genomic instability [42]. This finding links DNA-damage to apoptosis possibly *via* caspase-2 activation. Thus, caspase-2 appears to mediate functions in the cell nucleus (cell cycle checkpoint regulation) as well as apoptosis in response to appropriate stimuli which may occur in the cytoplasm [25], [26]. Different function and cell distribution for the same protein is not unique as β-catenin is known to act as an integral cell-cell adhesion adaptor protein as well as a transcriptional co-regulator in the cell nucleus [43]. If caspase-2 only acts to mediate apoptosis through activation in the cytoplasm [25], [26] an important question is what signals lead to initiation of an apoptotic response through a shift of caspase-2 from the nucleus to the cytoplasm where oligomerization leading to activation occurs [25], [26].

In summary, we have found that pre-incubation of cells with NEM completely prevents an artifactual shift of nuclear caspase-2 as well as ERα to the extra-nuclear fraction when cells are lysed. This approach thus allows for a quantitative analyses of the kinetics of cell re-distribution of caspase-2 in response to apoptotic stimuli in relation to an apoptotic endpoint (e.g. TUNEL assay or AIF translocation from the mitochondria to the nucleus).
